# Circulating tumor DNA sequencing in colorectal cancer patients treated with first-line chemotherapy with anti-EGFR

**DOI:** 10.1038/s41598-021-95345-4

**Published:** 2021-08-11

**Authors:** Yoojoo Lim, Sheehyun Kim, Jun-Kyu Kang, Hwang-Phill Kim, Hoon Jang, Hyojun Han, Hyoki Kim, Min Jung Kim, Kyung-Hun Lee, Seung-Bum Ryoo, Ji Won Park, Seung-Yong Jeong, Kyu Joo Park, Gyeong Hoon Kang, Sae-Won Han, Tae-You Kim

**Affiliations:** 1 Department of Internal Medicine, Seoul National University Hospital, 101 Daehak-ro, Jongno-gu, Seoul, 03080 Republic of Korea; 2 Department of Molecular Medicine and Biopharmaceutical Sciences, Graduate School of Convergence Science and Technology, Seoul National University, Seoul, Korea; 3 Cancer Research Institute, Seoul National University, Seoul, Korea; 4 Celemics, Inc., Seoul, Korea; 5 Department of Surgery, Seoul National University Hospital, Seoul, Korea; 6 Department of Pathology, Seoul National University Hospital, Seoul, Korea

**Keywords:** Colorectal cancer, Tumour biomarkers

## Abstract

Circulating tumor DNA (ctDNA) may reveal dynamic tumor status during therapy. We conducted serial ctDNA analysis to investigate potential association with clinical outcome in metastatic colorectal cancer (mCRC) patients receiving chemotherapy. Tissue *KRAS*/*NRAS* wild-type mCRC patients were enrolled and treated with first-line cetuximab-containing chemotherapy. ctDNA isolated from plasma were analyzed by next generation sequencing (NGS) with 16 targeted gene panel. Among 93 patients, 84 (90.3%) had at least 1 somatic mutation in baseline ctDNA samples (average 2.74). Five patients with *KRAS* or *NRAS* hotspot mutation in the ctDNA showed significantly worse progression-free survival (PFS) (*p* = 0.029). Changes in average variant allele frequency (VAF) in ctDNA showed significant correlation with tumor size change at the time of first response evaluation (*p* = 0.020) and progressive disease (PD) (*p* = 0.042). Patients whose average VAF decreased below cutoff (< 1%) at the first evaluation showed significantly better PFS (*p* < 0.001), and the average VAF change further discriminated the PFS in the patients in partial response (*p* = 0.018). At the time of PD, 54 new mutations including *KRAS* and *MAP2K1* emerged in ctDNA. ctDNA sequencing can provide mutation profile that could better reflect tumor mutation status and predict treatment outcome.

## Introduction

Tumor DNA with specific genetic alterations is present as minor sub-clones in the cell-free fraction of the peripheral blood^1–3^. Analysis of the plasma circulating tumor DNA (ctDNA) is a non-invasive alternative to classical tissue-based analysis for tumor specific genetic alterations^4–6^. Not only non-invasive, but it may also provide better means of monitoring in light of spatial and temporal tumor heterogeneity. ctDNA analysis makes tumor analysis possible in the patients whose tissue specimens are otherwise impossible to obtain, for medical or anatomical reasons. And it makes it feasible to obtain multiple specimens repeatedly. Furthermore, as blood can carry ctDNA shed into tumors at different locations in the body, ctDNA may be more comprehensive representation of the tumor mutations of an individual patient^7,8^. Longitudinal ctDNA analysis can also provide a molecular profile of how a tumor evolves and changes over time in response to chemotherapy. Recently, ctDNA analysis is rapidly being implemented into clinical practice. However, the evidence is still not strong enough to apply ctDNA analysis to aid the actual clinical decisions^9^.

Recent advances in next generation sequencing (NGS) technology have enabled evaluation of various genetic variations in a single procedure. There have been explorations for application of NGS to ctDNA detection, and various methods from improvement in sample collections to deeper sequencing coverage, molecular barcoding methods and other error-suppressing algorithms have been studied to improve sensitivity and specificity^10,11^. Along with the advancement, we have previously developed and published a simple bioinformatic pipeline for discovering somatic mutations using NGS technology from ctDNA of patients with cancer^12^.

Cetuximab, an anti-EGFR monoclonal antibody, is one of the widely used drugs for first-line treatment of all-RAS wild type, metastatic colorectal cancer (mCRC) in combination with cytotoxic chemotherapy^13,14^. The test for *KRAS* and *NRAS*, has become a prerequisite for the treatment decision of mCRC patients, and the gold standard is to use tissue samples. However, tissue acquisition for molecular diagnosis before treatment decision is not possible in some of the mCRC patients. Moreover, there are still some patients who would not respond to cetuximab even after confirmation of RAS wild-type by conventional methods.

In this study, we aimed at evaluating the potential association of the results of ctDNA analysis with clinical outcome in metastatic cancer patients, and the feasibility of application of ctDNA analysis to actual patients. For the purpose, we prospectively registered tissue-proven RAS wild-type metastatic colorectal cancer patients and collected blood samples before, during and after palliative 1st line chemotherapy with cetuximab-containing regimen.

## Methods

### Patients and treatment

Adult patients with pathologically confirmed diagnosis of all-RAS wild type metastatic colorectal cancer, for whom a decision has already been made to be treated with 1st line palliative chemotherapy with cetuximab-containing regimen at Seoul National University Hospital (SNUH) were enrolled in this study. Mutation status of *KRAS* exon 2,3,4 and *NRAS* exon 2,3,4 were determined before enrollment using direct DNA sequencing of tissue samples to the product of nested-PCR on specific exon (N = 88) or targeted gene sequencing with NGS platform (N = 5), as part of standard-of-care testing at SNUH. The chemotherapy backbone of the cetuximab-containing regimen was chosen between FOLFIRI (5-Fluorouracil, Leucovorin, Irinotecan) or FOLFOX (5-Fluorouracil, Leucovorin, Oxaliplatin), at the discretion of the treating physician. Response evaluation was done in accordance to RECIST 1.1^15^ using contrast-enhanced computed tomography (CT) obtained at baseline and repeated every four cycles or at clinician’s suspicion of progressive disease.

All patients provided written informed consent before any study-specific procedures. The protocol of this study was reviewed and approved by the Institutional Review Board (IRB) of SNUH (IRB number: 1509-095-705) and was conducted in accordance with the Declaration of Helsinki in biomedical research involving human subjects.

### Blood sample collection and cell-free DNA extraction

Serial blood samples were obtained before treatment initiation (≤ 7 days before treatment) and at the time of response evaluations. Whole blood (8–10 mL) was collected into EDTA tubes during routine phlebotomy. Blood samples were centrifuged with Ficoll solution at 1500 × *g* for 15 min. Plasma was then separated by centrifugation at 16,000 × *g* for 10 min to remove cell debris, after which 1-mL aliquots were placed in Eppendorf tubes and stored at − 80 °C before extraction. This protocol was performed within 20 min of blood collection to prevent cell-free DNA (cfDNA) degradation and release of genomic DNA from dying blood cells. cfDNA was isolated according to the manufacturer’s instructions from 2 to 4 mL plasma using a cfKapture™ Kit (MagBio Genomics, USA) and quantified using a 2200 TapeStation (Agilent Technologies, Santa Clara, CA, USA). Peripheral blood mononuclear cell (PBMC) were separated following this protocol. Genomic DNA was isolated from PBMC using a QIAamp DNA Mini Kit (Qiagen).

### Tumor tissue sample preparation

We used archival tissue samples from patients included in the study, when available. Study samples included formalin-fixed, paraffin-embedded (FFPE) and fresh frozen tissues collected at the time of surgical resection. Genomic DNA was isolated from each sample using a Qiagen DNA FFPE Tissue Kit (Qiagen, Hilden, Germany) for FFPE samples and a ReliaPrep gDNA Tissue Miniprep System (Promega) for fresh-frozen tissues. After isolation, the concentrations and purities of genomic DNA were measured using a spectrophotometer (ND1000; Nanodrop Technologies, Thermo Fisher Scientific, MA, USA).

### Targeted deep sequencing for multiple gene panel

Blood and tissue samples from patients with mCRC were analyzed using ultra-deep targeted sequencing for two versions of panel including genes frequently mutated in CRC and are related to sensitivity to EGFR blockade. Panel ver. 2 (*APC*, *TP53*, *KRAS*, *PIK3CA*, *BRAF*, *EGFR*, *ERBB2*, *ERBB3*, *FGFR1*, *NRAS*, and *HRAS*—total 11 genes) was applied for 88 samples and panel ver. 3 (*APC*, *TP53*, *KRAS*, *PIK3CA*, *BRAF*, *EGFR*, *ERBB2*, *ERBB3*, *FGFR1*, *NRAS*, *HRAS*, *IRS1*, *MAP2K1*, *MET*, *PDGFRB*, and *PTEN*—total 16 genes) was applied for 304 samples (Supplementary Table 1). A DNA NGS library was constructed using a Celemics NGS DNA Library Prep Kit. For cfDNA sequencing, a random barcode was introduced into P7 index sites to sort out true variant reads and discard sequencing errors. Also, in-house scripts were used for identifying tagged barcodes and removing duplicated reads to correct bias errors on calculating allele frequency, which were frequently caused by PCR duplicates.

Solution-based target enrichment was performed at Celemics, Inc. (South Korea), using a custom target capture panel. Captured DNA libraries were sequenced using an Illumina HiSeq 2500 platform (Illumina, San Diego, CA, USA) in 2 × 150 bp paired-end mode.

### NGS data analysis: sequence alignment and variant calling

All sequencing reads from the samples were generated as fastq format. Filtered fastq files were aligned to the human reference genome (hg19) using Burrows–Wheeler Aligner (v0.7.10) “mem” algorithm. Aligned SAM files were converted into BAM files and sorted using SAMtools (v1.1). Local realignment around known indel sites and base quality score recalibration were performed with GATK (v4.1.0.0). After generating pileup files with SAMtools mpileup, variants were called using Varscan2^16^. Genetic variants were annotated using SnpEff^17^ and variant information from public databases including ClinVar (https://www.ncbi.nlm.nih.gov/clinvar/) and COSMIC (https://cancer.sanger.ac.uk/cosmic) was added by our in-house scripts. We constructed a sequential bioinformatic pipeline for these analyses and executed it for all samples in the same way (Supplementary Table 2).

### Analysis of genetic alterations from cfDNA, Tissue and PBMC

In our study, we chose single nucleotide variants (SNV), and insertion/deletion variants (Indel) that were classified into missense, stop-gain, stop-loss, in-frame deletion and frameshift mutations to collect possibly functional mutations. For analysis of variants from biopsied or surgical resected tissue, we filtered out variants which have less than 10% of variant allele frequency (VAF) or have less than 10 variant reads to remove false positives or sequencing errors. In case of cfDNA data, we took variants that have at least 1% of VAF and 10 variant reads, considering small amount of ctDNA in plasma cfDNA (Supplementary Fig. 1). Next step, we used mutations from PBMC samples to sort out genomic variants and leave only somatic mutations. We listed up PBMC variants with more than 20% of VAF as germline variants (most are around 50% or 100% VAF corresponding to heterozygote or homozygote mutation, respectively) and removed these variants from results of cfDNA and tissue in the same patient. On the other hand, we gathered PBMC variant calls which had less than 20% VAF from all PBMC samples. These variants comprised a set of blacklist alterations which were assumed to repeatedly occurring sequencing errors or noise variants including clonal hematopoiesis. We lastly filtered out additional error or noise with visual inspection using the IGV browser^18^ and finalized the tumor driven mutations from each cfDNA or tissue sample (Supplementary Fig. 1).

### Statistical analysis

In patients with wild-type *KRAS* and *NRAS* CRC, the estimated frequency of mutations in *EGFR*, *ERBB2*, *ERBB3*, *FGFR1*, *KRAS*, *NRAS*, *HRAS*, *BRAF*, and *PIK3CA* genes is around 40% (http://www.cbioportal.org/public-portal/). At the prevalence of 40%, a total sample size of 78 could achieves 80% power to prove improvement of sensitivity on mutation detection from 0.7 to 0.9 using a two-sided binomial test^19^. The target significance level is 0.05. When we assume a test failure rate of 10%, the required sample size is equal to 87 and we satisfy the requirement with enrollment of total 93 patients. Sample size calculation was made by Power Analysis and Sample Size software (PASS program for Windows; http://www.ncss.com).

Nonparametric test, Mann–Whitney U test was applied for comparing the largest tumor size, serum carcinoembryonic antigen (CEA) and cfDNA concentrations of patients with or without baseline ctDNA mutation. PFS is defined as the time from the start of chemotherapy to date of disease progression or death from any cause. Patients without progression or death at the time of data collection will be censored at the date last known to be alive and progression free. Kaplan–Meier method with log rank test was applied for PFS analysis and survival curve plot. Chi-square test and Fisher’s exact test were used to evaluate the response rate of chemotherapy between ctDNA mutant patients and wild type patients. Pearson correlation for parametric test and Spearman’s rank correlation for the nonparametric test was used to compare changes in average VAF and changes in target tumor lesion size. All analysis was performed using the Statistical Package for the Social Sciences software (IBM^®^ SPSS^®^ statistics, version 25.0).

## Results

### Patient characteristics

A total of 93 metastatic colorectal cancer patients were enrolled in this study. Patient characteristics are summarized in Table 1. 71 patients (76.3%) had metastatic lesions in liver, 21 (22.6%) in lung and 20 (21.5%) had peritoneal carcinomatosis while 20 patients (21.5%) had metastatic lesions in multiple organs. 75 (79.6%) patients received cetuximab plus FOLFIRI and 18 (19.4%) patients received cetuximab plus FOLFOX.

**Table 1:** Baseline characteristics (n = 93).

Categories	Number of patients (%)
Age, median (range)	61 (26–80)
**Sex**	
Male	70 (75.3%)
Female	23 (24.7%)
**Disease presentation at enrollment**	
Metastatic	74 (79.6%)
Recurrent	19 (20.4%)
**Primary tumor site**	
Ascending colon	13 (14.0%)
Transverse colon	1 (1.1%)
Descending colon	5 (5.4%)
Sigmoid colon	48 (51.6%)
Rectum	26 (28.0%)
**Metastasis site**	
Liver	71 (76.3%)
Lung	21 (22.6%)
Peritoneal seeding	20 (21.5%)
Lymph nodes	27 (29.0%)
Other organs	9 (9.7%)
**Cytotoxic chemotherapy**	
FOLFIRI	75 (80.6%)
FOLFOX	18 (19.4%)
**Pathology**	
ADC, W/D	3 (3.2%)
ADC, M/D	73 (78.5%)
ADC, P/D	12 (12.9%)
Signet ring cell	3 (3.2%)
metastatic ADC	2 (2.2%)
**Microsatellite instability**	
MSS	78 (83.9%)
MSI-L	10 (10.7%)
MSI-H	1 (1.1%)
Unknown	4 (4.3%)

*FOLFIRI* 5-Fluorouracil + Leucovorin + Irinotecan, *FOLFOX* 5-Fluorouracil + Leucovorin + Oxaliplatin, *ADC* adenocarcinoma, *W/D* well differentiated, *M/D* moderately differentiated, *P/D* poorly differentiated, *MSS* microsatellite stable, *MSI-L* microsatellite instability—low, *MSI-H* microsatellite instability—high.

92 patients data were available for response evaluation. 1 patient discontinued study participation before the first response evaluation at patient’s will. The best responses in the 92 patients were partial response (PR) in 69 (75.0%), stable disease (SD) in 19 (20.7%) and progressive disease (PD) in 4 (4.3%). Blood samples at disease progression were available from 54 patients. The median progression-free survival (PFS) of all patients was 10.5 months.

### Pre-treatment tumor mutation profile in ctDNA

Among the 93 baseline plasma samples, we identified a total of 230 mutations in 84 (90.3%) samples (Supplementary Table 3). Patients with mutations had an average of 2.74 mutations. Frequently mutated genes are shown in Table 2. Most patients had mutations on *APC* (77.4%) or *TP53* (68.8%) genes. Although RAS wild-type by tissue was prerequisite for enrollment, 7 *KRAS* mutations and 2 *NRAS* mutations were found in baseline ctDNA samples of 7 patients (1 patient had 2 distinct *KRAS* mutations and another 1 patient had both 1 KRAS and 1 NRAS mutations), 5 of which were hotspot mutations [*KRAS* G13D (VAF 2.1%), G12D (VAF 9.5%, 1.4%, and 1.1%) and *NRAS* Q61R (VAF 1.3%)].

**Table 2:** Frequency of baseline ctDNA mutations.

Gene	No. mutations	No. patients with mutations (%)
APC	111	72 (77.4%)
TP53	71	64 (68.8%)
ERBB2	13	9 (9.7%)
PTEN^a^	6	5 (7.2%)
KRAS	7	6 (6.5%)^b^
PIK3CA	6	6 (6.5%)
MET^a^	4	3 (4.3%)
PDGFRB^a^	3	3 (4.3%)
BRAF	3	3 (3.2%)
NRAS	2	2 (2.2%)^b^
ERBB3	2	2 (2.2%)
MAP2K1^a^	1	1 (1.4%)
EGFR	1	1 (1.1%)
Total	230	

^a^Genes included only in panel version 3 used for baseline ctDNA analysis of 69 patients.

^b^1 patient had 2 distinct KRAS mutations and another 1 patient had both 1 KRAS and 1 NRAS mutations.

No mutation was found from 9 (9.7%) patients. The tumor size, compared by the largest diameter of the largest measurable lesion, was significantly smaller in patients without any ctDNA mutations compared to those with mutations (median 17 (95% CI 10–20, N = 7) vs. 35 (95% CI 29–42, N = 79) mm, respectively; *p* < 0.001, Supplementary Fig. 2a). Serum CEA was also lower in patients without any mutations compared to ctDNA mutation positive patients (median 4.6 (95% CI 0.5–18.0, N = 9) *vs.* 35.8 (95% CI 14.4–49.3, N = 82) ng/mL, respectively; *p* = 0.006). Furthermore, difference in the pattern of metastasis was observed between the patients with or without mutations. Patients with liver metastasis (N = 71) were more likely to have mutations detected in ctDNA (ctDNA mutation detected in 95.8% (68/71) of patients with liver metastasis vs. 72.7% (16/22) in patients without liver metastasis, *p* = 0.005), while patients with peritoneal seeding without any other distant metastasis (N = 7) were less likely to have mutations detected in ctDNA (ctDNA mutation detected in 42.9% (3/7) of patients with peritoneal seeding only vs. 94.2% (81/86) of patients with any metastatic lesions other than peritoneal seeding, *p* = 0.001). There was no significant difference in cfDNA concentration according to ctDNA mutation status (*p* = 0.33, Supplementary Fig. 2b).

Archival tissue samples from 66 patients were available for targeted sequencing. 46 were FFPEs, and 20 were fresh-frozen tissues. A total of 137 mutations were found from 58 patients and used to compare against the ctDNA mutations found from the same patients (Supplementary Table 4). The mutations found from tissue samples were covered by ctDNA in 50 of the 66 patients (75.8%). Comparing by each mutation, 97 (70.8%) of 137 tissue variants were matched with ctDNA mutations. 34 (85.0%) of the 40 unmatched tissue variants were observed in ctDNA at minor allele frequencies below our cutoff level (< 1%). Meanwhile, we identified 44 ctDNA mutations which were not detected from tissue samples. 31 (70.5%) of the 44 ctDNA only mutations were observed at minor sequencing reads below our cutoff.

### Association of baseline ctDNA mutations with treatment outcome

**Figure 1:**
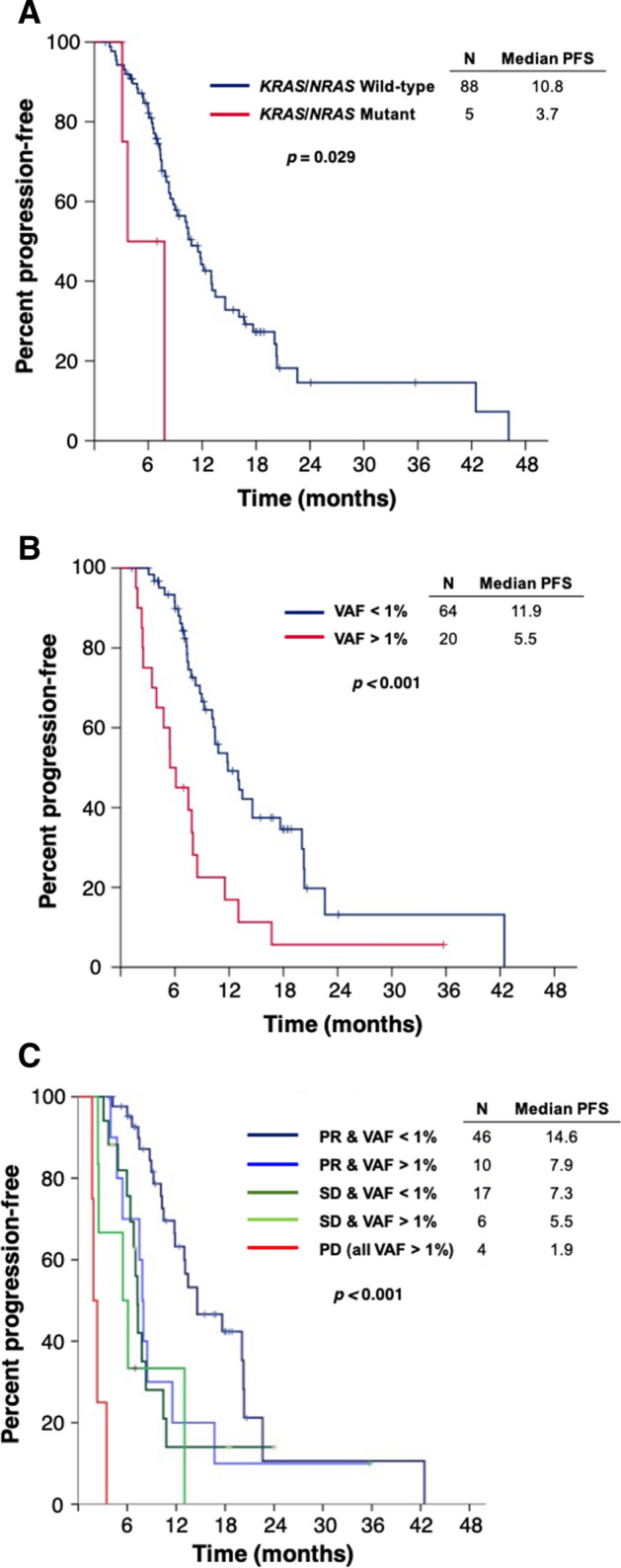
Treatment response related to baseline ctDNA mutations. (**A**) Kaplan–Meier curves of PFS according to baseline *KRAS* or *NRAS* hotspot mutations by ctDNA. (**B**) Kaplan–Meier curves of PFS according to average VAF changes after treatment. (**C**) Kaplan–Meier curves of PFS according to average VAF changes and RECIST 1.1 response groups at the time of first response evaluation (log-rank *p*-value for all groups).

We analyzed the treatment outcome in relation to baseline ctDNA mutations that have been labeled pathogenic or likely-pathogenic by ClinVar database^20^. The PFS of the patients with pathogenic hotspot *KRAS* or *NRAS* mutations (N = 5) in baseline ctDNA was significantly shorter (*p* = 0.029, median PFS 3.7 months in mutants vs. 10.8 months in wild type, Fig. 1A). These *KRAS/NRAS* mutant patients showed tendency towards lower response rate (40.0% in mutants vs. 77.1% in wild type, *p* = 0.098). Among the ctDNA *KRAS*/*NRAS* wild type patients, patients with *BRAF* or *MAP2K1* mutations (N = 3) showed trend towards shorter PFS (*p* = 0.14, median PFS 4.8 months in mutants vs. 10.8 months in wild type).

### Changes in ctDNA mutations and tumor response

The average VAF for all ctDNA mutations were calculated for each patient at each time point. The average VAFs from baseline ctDNA of all patients with measurable lesions showed positive correlation between average VAFs and the size of the largest tumor lesion measured by CT (Pearson correlation coefficient *r* = 0.607, *p* < 0.001, Supplementary Fig. 3a). These average VAFs of ctDNA mutations could better represent the tumor burden than cfDNA concentrations or values of average VAF multiplied by cfDNA concentrations (Supplementary Fig. 3b,c).

**Figure 2:**
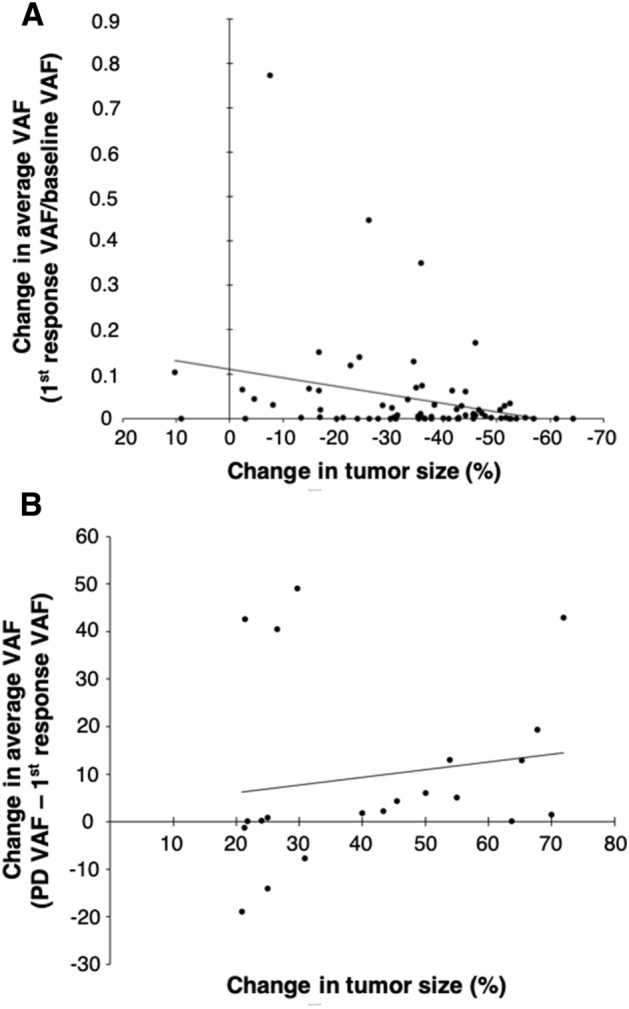
Correlation between changes of ctDNA mutation VAF and tumor size. (**A**) Correlation of average VAF changes and tumor size changes between baseline and first evaluation. (**B**) Correlation of average VAF changes and tumor size changes between first evaluation (or baseline if confirmed PD at the first evaluation) and at the time of disease progression.

The mean of average VAF of all 84 patients at baseline was 23.34% and it decreased to less than 1% after chemotherapy in 76.2% of patients. The change of average VAF between the baseline and the first response evaluation showed a linear correlation with tumor size changes on CT images (Pearson’s *r* = − 0.27, *p* = 0.020, Fig. 2A). Patients whose average VAF decreased below cutoff level (< 1%) at the first evaluation (N = 64) showed significantly better PFS compared to patients with higher average VAF (N = 20) (*p* < 0.001, median PFS 11.9 months in VAF < 1% vs. 5.5 in VAF > 1%; Fig. 1B). At the time of first evaluation, the patients who were in PR by RECIST 1.1 but had higher average VAF (> 1%) showed significantly poorer PFS compared to the other patients in PR (*p* = 0.018, median PFS 14.6 months in VAF < 1% vs. 7.9 in VAF > 1%), and the PFS curve of those 10 patients was similar to the patients in SD (Fig. 1C). Among the 54 patients with blood samples at the time of disease progression, there was also a significant increase in the mean of average VAF at disease progression (*r* = 0.45, *p* = 0.042, Spearman’s rank correlation, Fig. 2B).

### Detection of resistance mutations in ctDNA

To identify the mutations related to resistance to cetuximab, we have compared the mutations of ctDNA from the blood samples at confirmed PD with the mutations from tissue samples and blood samples acquired at other time points. From the 54 patients with blood samples available at the time of PD, we were able to find 54 new mutations from the ctDNA at the time of resistance.

The 54 new mutations included variants of *KRAS*, *NRAS* and *MAP2K1* which are components of *RAS/MAPK* signaling pathway (Table 3). Among the 54, 11 (20.4%) were pathogenic or likely pathogenic and 5 (9.3%) were of uncertain significance by ClinVar database^20^ (Supplementary Fig. 4). Of the mutations, *KRAS* G12C, G12V, Q61H and *MAP2K1 K57T, K57N* were previously reported as resistance mutations after anti-EGFR inhibitor treatment (Table 4).

**Table 3:** Newly detected ctDNA mutations only at the time of disease progression.

Gene	No. mutations	No. patients with mutations (%)
APC	10	7 (14.9%)
TP53	9	7 (14.9%)
ERBB2	8	6 (12.8%)
KRAS	5	5 (10.6%)
PIK3CA	5	2 (4.3%)
MAP2K1^a^	4	4 (13.8%)
ERBB3	3	3 (6.4%)
MET^a^	3	1 (3.4%)
PDGFRB^a^	2	2 (6.9%)
EGFR	2	2 (4.3%)
PTEN^a^	1	1 (3.4%)
NRAS	1	1 (2.1%)
HRAS	1	1 (2.1%)
Total	54	

^a^Genes included only in panel version 3 used for PD ctDNA analysis of 34 patients.

**Table 4:** List of candidate resistance mutations arisen during anti-EGFR treatment. The variants were previously known for pathogenic or likely pathogenic mutations by ClinVar database.

ID	Chr	Start	End	Ref	Alt	Functional	Gene	Base change	AA change	VAF (%)	ClinVar	dbSNP
CTDC05	12	25398284	25398284	C	A	missense_variant	KRAS	c.35G > T	p.Gly12Val	1.74	Pathogenic/Likely_pathogenic	rs121913529
CTDC07	15	66727455	66727455	G	T	missense_variant	MAP2K1	c.171G > T	p.Lys57Asn	3.05	Pathogenic/Likely_pathogenic	NA
CTDC08	17	7577548	7577548	C	T	missense_variant	TP53	c.733G > A	p.Gly245Ser	15.27	Pathogenic/Likely_pathogenic	rs28934575
CTDC14	12	25380275	25380275	T	G	missense_variant	KRAS	c.183A > C	p.Gln61His	4.09	Pathogenic/Likely_pathogenic	rs17851045
CTDC41	5	112175639	112175639	C	T	stop_gained	APC	c.4348C > T	p.Arg1450*	38.41	Pathogenic/Likely_pathogenic	rs121913332
CTDC42	12	25398284	25398284	C	A	missense_variant	KRAS	c.35G > T	p.Gly12Val	3.61	Pathogenic/Likely_pathogenic	rs121913529
CTDC50	15	66727454	66727454	A	C	missense_variant	MAP2K1	c.170A > C	p.Lys57Thr	13.72	Likely_pathogenic	NA
CTDC52	10	89711900	89711900	G	A	missense_variant	PTEN	c.518G > A	p.Arg173His	1.4	Pathogenic/Likely_pathogenic	rs121913294
CTDC70	12	25398285	25398285	C	A	missense_variant	KRAS	c.34G > T	p.Gly12Cys	2.44	Pathogenic/Likely_pathogenic	rs121913530
CTDC71	12	25398284	25398284	C	A	missense_variant	KRAS	c.35G > T	p.Gly12Val	1.92	Pathogenic/Likely_pathogenic	rs121913529
CTDC88	5	112177901	112177901	C	T	stop_gained	APC	c.6610C > T	p.Arg2204*	2.95	Likely_pathogenic	NA

## Discussion

In this study, we have collected serial ctDNA samples from mCRC patients at the start, in the middle, and the end of chemotherapy and analyzed mutation profiles of the ctDNA samples by applying targeted deep sequencing with NGS platform. ctDNA analysis was able to detect baseline mutations related to resistance to anti-EGFR treatment, and serial analysis have shown that changes in average VAF in ctDNA were associated with the response to treatment and have revealed a few emerging mutations at the time of resistance. Moreover, ctDNA analysis was able to detect additional *KRAS* and *NRAS* mutations that were not detected by conventional tumor tissue analysis, and the cases with newly detected RAS mutations showed poor response to cetuximab-containing chemotherapy.

With the recent advances in cancer diagnosis and treatments, tumor molecular profiling has become indispensable part of cancer treatment decision process. As obtaining tumor tissues for molecular profiling requires surgical resection or invasive biopsy, less invasive liquid-biopsy, especially collecting ctDNA from blood samples is emerging as an attractive alternative. With the potential advantages and the advancement in technologies, there has been increased interest in the application of ctDNA to the care of cancer patients and the utility is being evaluated in avid manner. Various research on clinical utilities ranges from utilizing ctDNA for detection of driver and resistance mutations for drug selection, and prediction and tracking of tumor burden and response to treatment including atypical responses such as immunotherapy^4,21–23^.

There are variabilities of ctDNA detection among individuals with cancer, even in patients with advanced disease and even among patients with the same cancer type^2^, and the relationship between the tumor biology and the release of ctDNA is still not well understood. In advanced colorectal cancer, previously reported fractions of patients with detectable ctDNA have been approximately 85%^24,25^, which is relatively higher than those with other tumor types. In our report, 90.3% of the included patients had at least 1 somatic mutation detected from ctDNA. All our patients had confirmed metastatic disease, and all of our baseline samples were collected before the initiation of any palliative chemotherapy, important for obtaining high yield. Furthermore, our results suggest that patients with larger metastatic lesions, especially in sites where it is assumed to have spread hematogenously, are more likely to have mutations detected by ctDNA. This may be related to the difference in mode of spread, affecting the amount of ctDNA shed into blood stream. However, because our gene panel was not aimed to completely cover all frequently mutated genes in CRC but was targeted on covering EGFR pathway related genes, differences in biology may have contributed to differences in detection of mutations across different metastatic patterns.

Nonetheless, we were able to identify the mutations already well known to be associated with cetuximab-resistance by ctDNA and show their relevance to cetuximab sensitivity. Previously known pathogenic mutations in EGFR pathway in baseline ctDNA were predictive of response to a cetuximab plus chemotherapy regimen and they also emerged as resistance mutations at the time of progression. More importantly, we were able to discover 7 (7.5%) *KRAS* and *NRAS* mutations in 93 patients, demonstrated to have RAS wild-type tumors, from tissue by conventional methods. There are prior studies on concordance of ctDNA based RAS testing in CRC compared to tissue RAS testing using digital PCR^26^, BEAMing^27,28^ or NGS^29,30^. Overall specificity of ctDNA RAS mutation detection compared to tumor tissue in these studies was 0.91^31^, suggesting that approximately 9% of RAS wild-type cases by tissue analysis could be RAS mutant by ctDNA analysis, which is comparable to our result of 7.5%.

The *KRAS* and *NRAS* mutations are known to be the most important in predicting the sensitivity to anti-EGFR treatment in mCRC patients. *KRAS* is known to play an important role early in colorectal carcinogenesis, and the mutation status of *KRAS* between primary and metastatic sites have been reported to be mostly concordant. However, the existence of intraindividual heterogeneity has been reported in multiple studies. In one study, the heterogeneity between primary tumor and lymph node was reported to be up to 31% and that between primary tumor and distant metastasis was up to 10%^32^, and a meta-analysis reported similar results^33^. In our study, the tissue analysis in the 7 patients with ctDNA RAS mutations were all done with the primary tissues, by direct sequencing in 5 and by NGS platform in 2. Although we did not have metastatic tumor tissues to confirm the spatial heterogeneity of RAS mutations in these patients, the fact that these mutations included hotspots and that these patients with hotspot ctDNA RAS mutations exhibited significantly poor PFS supports that ctDNA was able to capture the RAS mutations missed from conventional analysis with tissue obtained from only one anatomical location. One of the 7 patients had multiple primary tumors in colon, a T4 lesion in sigmoid colon and a T3 lesion in transverse colon, and the T4 lesion was used to assess the mutational status of RAS for enrollment in this study. After ctDNA analysis, we retrospectively checked the RAS status of the smaller tumor and found the same KRAS mutation found in ctDNA. These results suggest that ctDNA may be better than classical tissue-based analysis in overcoming spatial heterogeneity for selecting patients who would benefit from EGFR blockade.

Many prior studies have sought for association between changes in ctDNA level and treatment response in patients with metastatic cancer undergoing systemic therapy^34–37^. As in other studies, our study results showed positive correlation between ctDNA changes and treatment outcome. Most other studies using a targeted sequencing method, measured somatic VAF and tracking mutations with highest VAF is one of reasonable surrogates for quantification of ctDNA^21^. In this study, we have chosen to compare the average of VAF of all mutations detected at each time point. As our treatment included both cytotoxic and targeted therapy, we assumed there would be clonal changes by selective pressure and calculated average of mutations to consider in the changes in mutations with highest frequency by clonal change. However, the best method of quantifying ctDNA to measure tumor burden for monitoring of treatment response has not been established^9^, and optimal methodology is yet to be determined.

Extracting true tumor derived mutations from all ctDNA variants is another hurdle to liquid biopsy. We used PBMC as controls to exclude germline mutations and variants arising from clonal hematopoiesis. Even after manual inspections to eliminate further noise, we had to apply a restrictive filter with VAF as there were still a substantial number of previously unreported minor mutations, which may be false positives. The application of unified filter for VAF with certain threshold did lead to decrease in sensitivity. In our study, 85% of the mutations identified from tissue samples that were not matched by ctDNA were found to have been filtered out from the ctDNA mutations list due to the VAF filter of 1%. However, most of hotspot mutations were not affected by the restrictive filter and considering the possibility of false positive calls affecting the clinical interpretation of mutational analysis, we decided to keep the filter at the threshold of 1%. Further optimization of analysis pipelines including noise reduction strategies may be necessary for improving sensitivity and specificity.

For this study, we examined only a limited number of genes that were already known to be related to EGFR pathway in mCRC. Moreover, we did not design our study to collect matching tumor tissues for all the included patients, or to obtain tissues around the time of blood sample collection. This may have limited us in making direct comparison of ctDNA to tissue-based analysis and from discovering novel ctDNA mutations related to anti-EGFR treatment sensitivity. We intended to focus on collecting practical evidence regarding feasibility of applying ctDNA analysis for treatment decisions. However, further study with matched tumor tissue collection and an advanced sequencing platform extending to more oncogenic genes may be necessary.

In summary, detection of ctDNA RAS mutations otherwise undetected from conventional tissue sequencing suggests an advantage of applying ctDNA analysis in making treatment decisions. Changes in ctDNA mutation show significant correlation with tumor response, and analysis of PD samples can detect resistance mutations.

## Supplementary Information

Supplementary Figures.

Supplementary Table 1.

Supplementary Table 2.

Supplementary Table 3.

Supplementary Table 4.

Supplementary Table 5.

## Data Availability

The data that supports the findings of this study are included in the supplementary materials of this article. The raw sequencing data have been deposited with links to BioProject accession number PRJNA714799 in the NCBI BioProject database (https://www.ncbi.nlm.nih.gov/bioproject/).
