# Supplementary material for: Circulating tumor DNA sequencing in colorectal cancer patients treated with first-line chemotherapy with anti-EGFR
Source: Sci Rep. 2021 Aug 11;11:16333. doi: 10.1038/s41598-021-95345-4 (PMC8358023; doi:10.1038/s41598-021-95345-4)

Supplementary Materials

Circulating tumor DNA sequencing in colorectal cancer patients treated with first-line chemotherapy with anti-EGFR

Yoojoo Lim[1,*], Sheehyun Kim[1,*], Jun-Kyu Kang[2,3,*], Hwang-Phill Kim[2], Hoon Jang[4], Hyojun Han[4], Hyoki Kim[4], Min Jung Kim[5], Kyung-Hun Lee[1], Seung-Bum Ryoo[5], Ji Won Park[5], Seung-Yong Jeong[5], Kyu Joo Park[5], Gyeong Hoon Kang[6], Sae-Won Han[1,3,**] and Tae-You Kim[1,2,3,**]

[1]Department of Internal Medicine, Seoul National University Hospital, Seoul, Korea

[2]Department of Molecular Medicine and Biopharmaceutical Sciences, Graduate School of Convergence Science and Technology, Seoul National University, Seoul, Korea

[3]Cancer Research Institute, Seoul National University, Seoul, Korea

[4]Celemics, Inc., Seoul, Korea

[5]Department of Surgery, Seoul National University Hospital, Seoul, Korea

[6]Department of Pathology, Seoul National University Hospital, Seoul, Korea

[*]These authors contributed equally to this work

Supplementary Table 1. Summary of collecting samples
Supplementary Table 2. Summary of panel sequencing statistics
Supplementary Table 3. 230 ctDNA mutations at baseline from total 93 patients
Supplementary Table 4. Tissue and baseline ctDNA mutations in 58 patients
Supplementary Table 5. 54 ctDNA mutations only detected at disease progression

Supplementary Figure. 1. Schematic diagram of the data analysis.

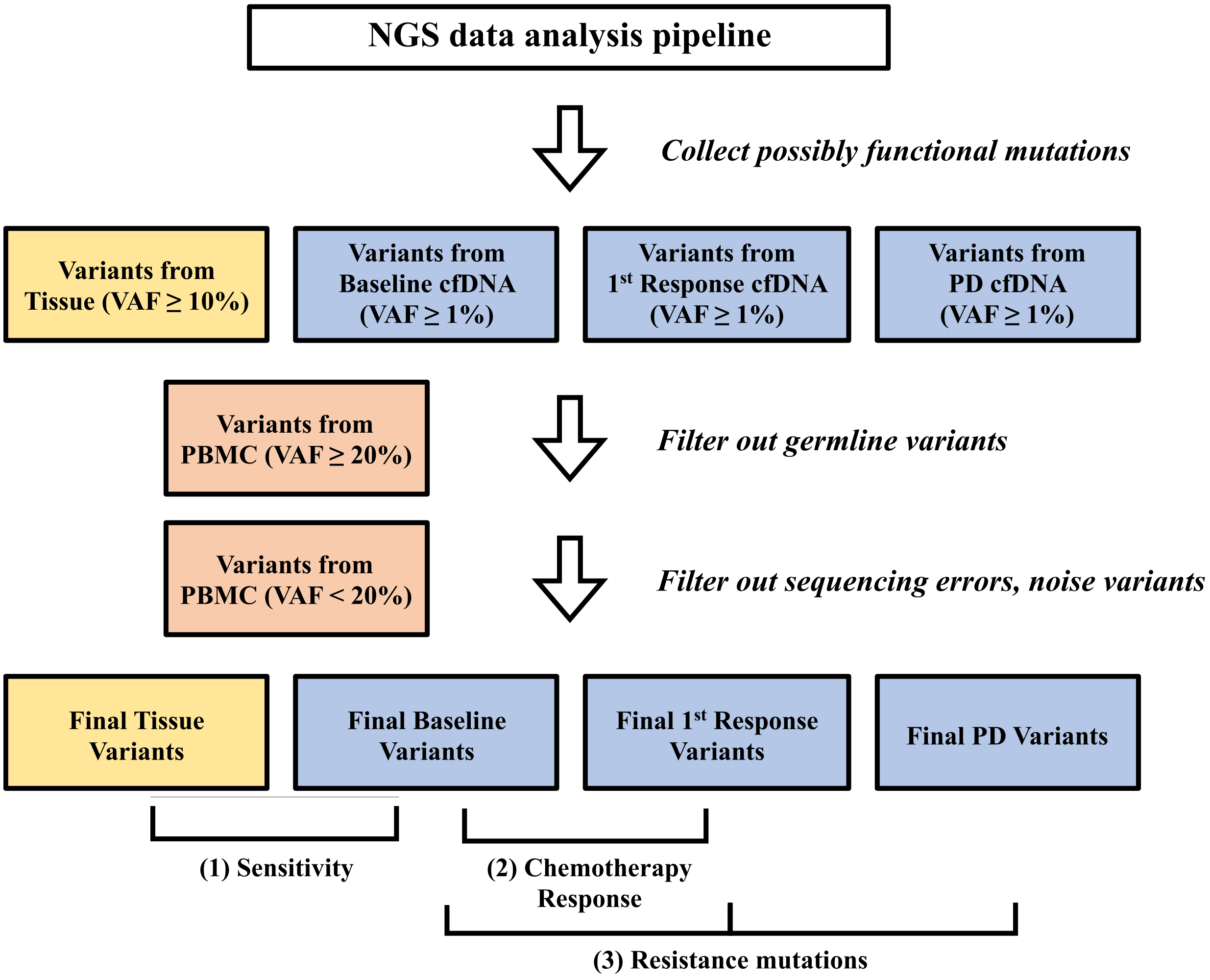

Supplementary Figure. 2. Comparison between detection of baseline ctDNA mutations for (a) the largest tumor size (mm) and (b) baseline cfDNA concentration (ng/ml).

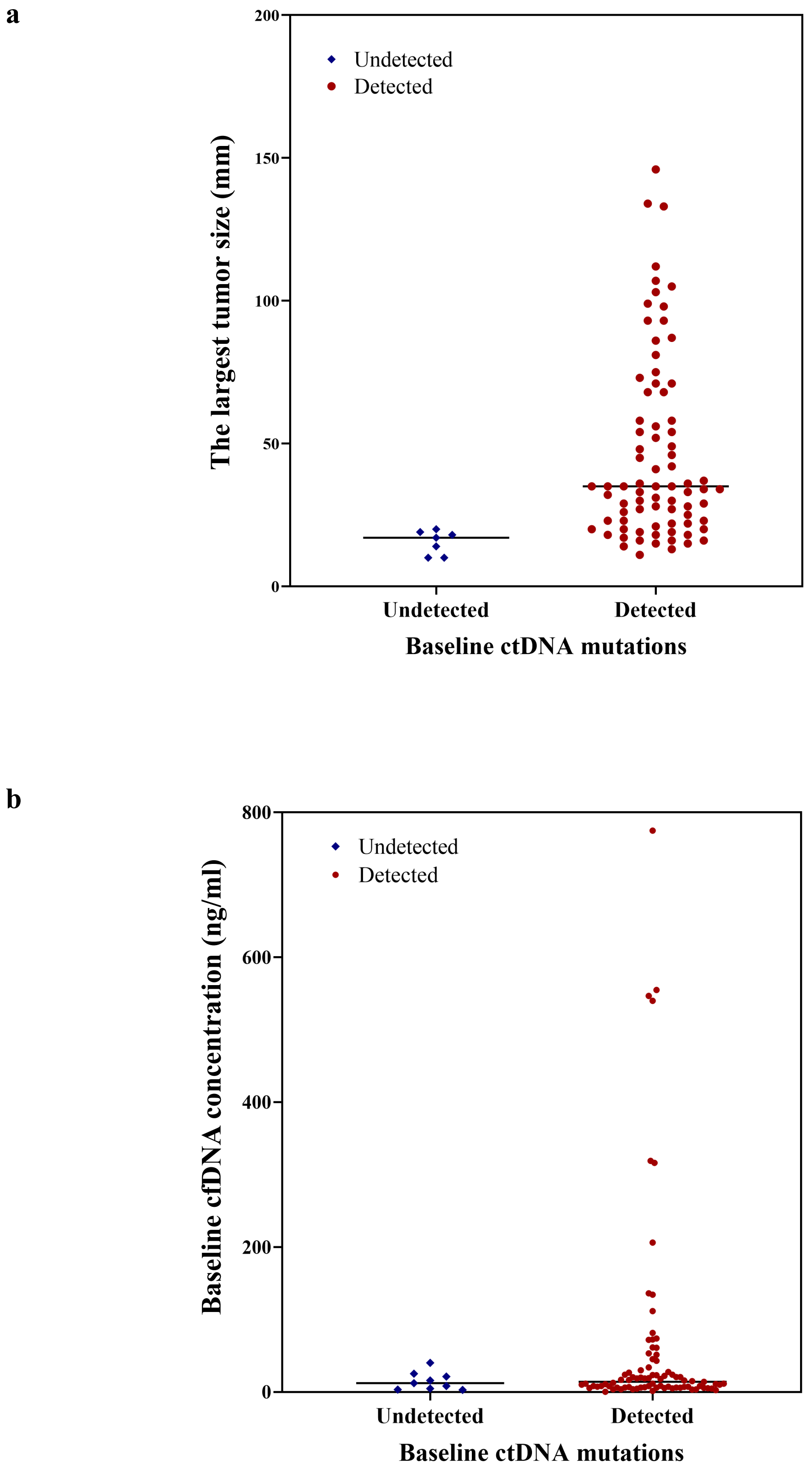

Supplementary Figure. 3. Correlation between tumor burden (defined as the largest target length, mm) and (a) baseline average VAF (%), (b) baseline cfDNA concentration (ng/ml) and (c) VAF (%) x cfDNA conc. (ng/ml).

a

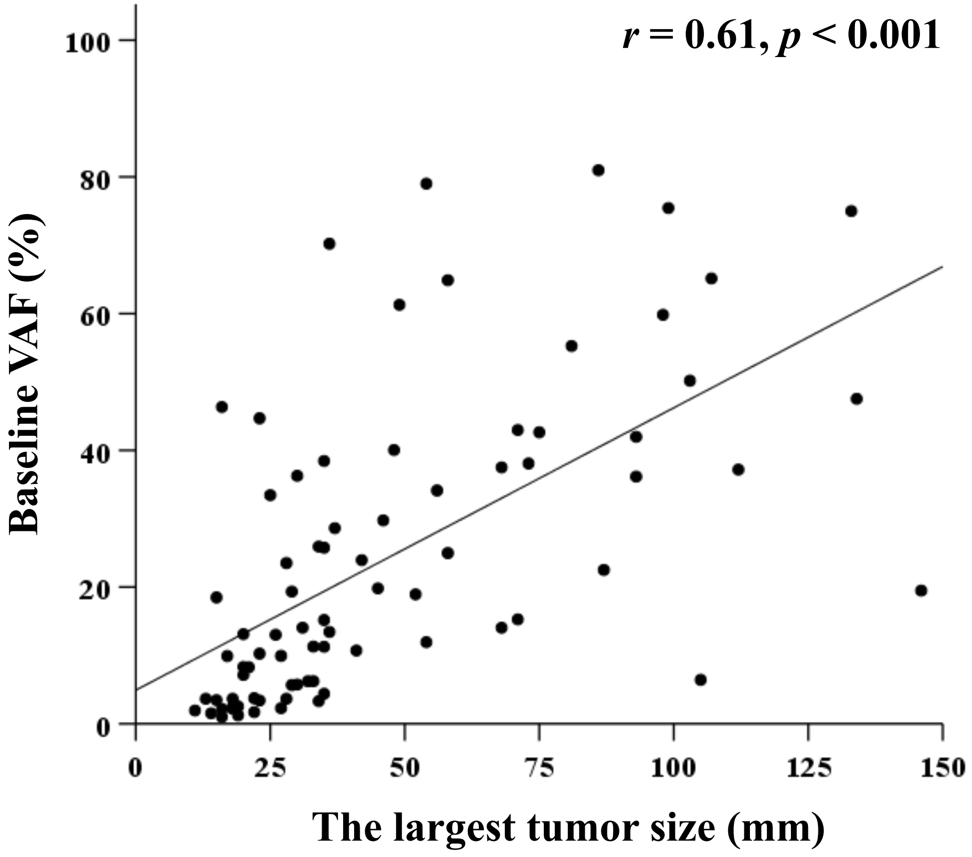

b

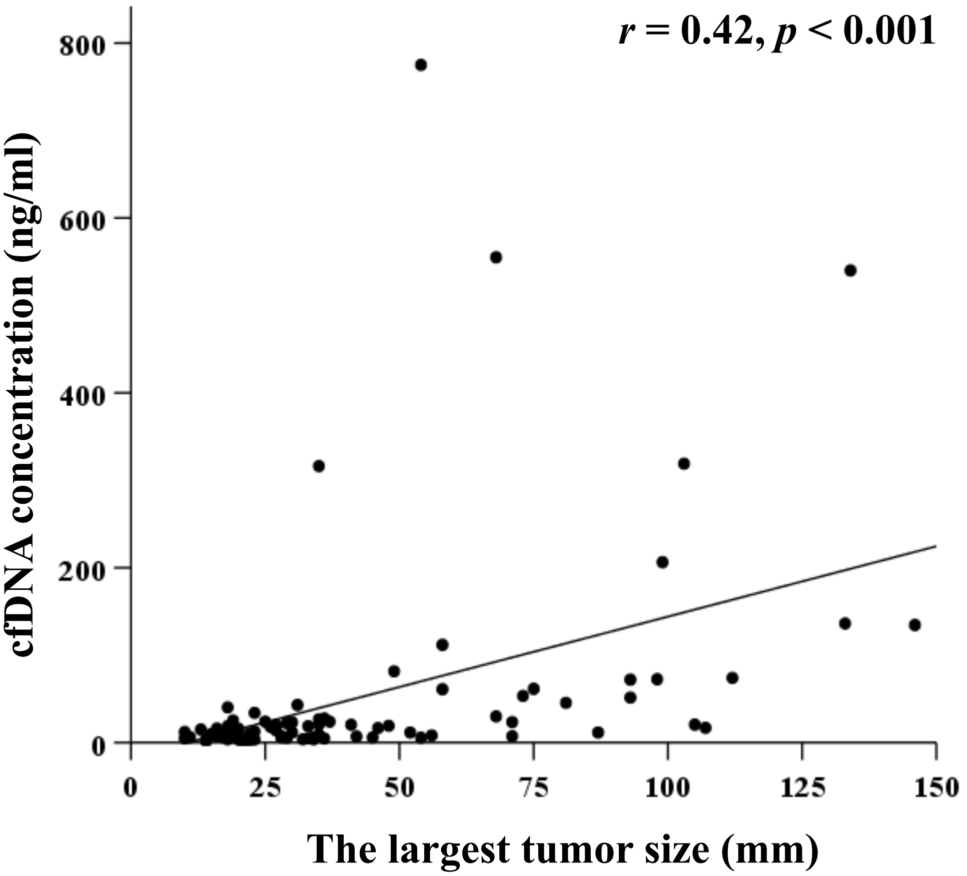

r = 0.42, p < 0.001
800
600
400
200
0
cfDNA concentration (ng/ml)
0
25
50
75
100
125
150
The largest tumor size (mm)

c

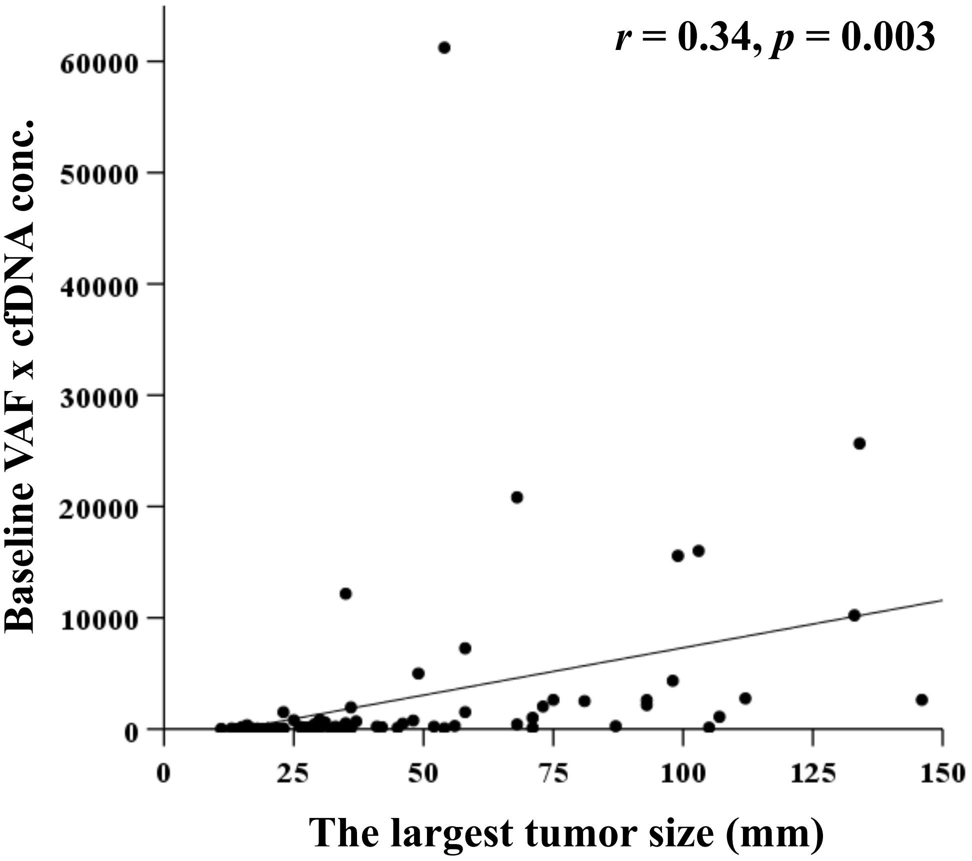

r = 0.34, p = 0.003
Baseline VAF x cfDNA conc.
60000
50000
40000
30000
20000
10000
0
0
25
50
75
100
125
150
The largest tumor size (mm)

Supplementary Figure. 4. Clinical significance of the mutations which were newly detected at disease progression by using annotation from the ClinVar database

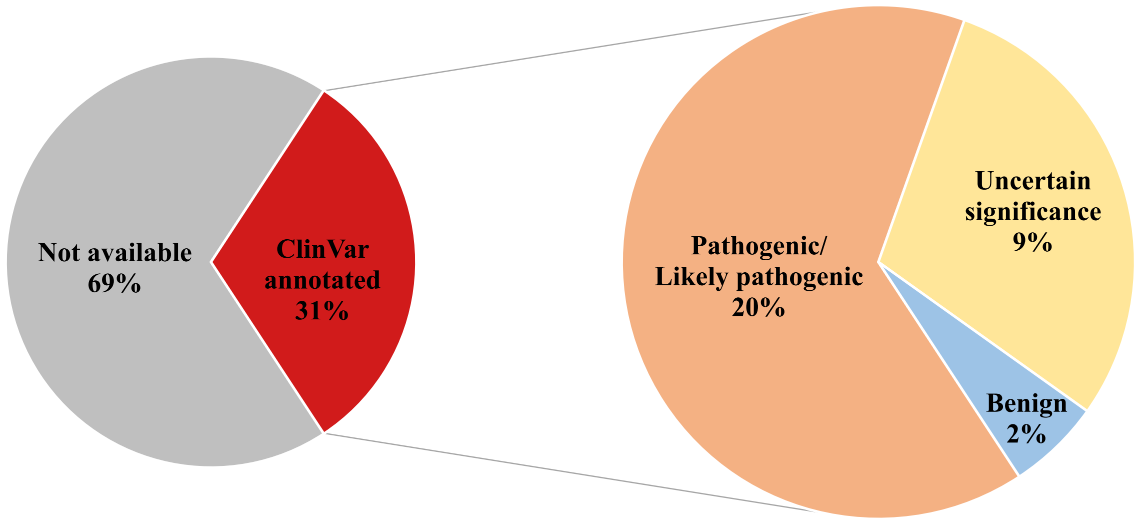

Supplement: Supplementary file 1 — Supplementary Figures. [file 41598_2021_95345_MOESM1_ESM.pdf]
